# Supplementary material for: The Evolution and Characterization of the RNA Interference Pathways in Lophotrochozoa
Source: Genome Biol Evol. 2024 May 7;16(5):evae098. doi: 10.1093/gbe/evae098 (PMC11114477; doi:10.1093/gbe/evae098)
Supplement: evae098_Supplementary_Data [file evae098_supplementary_data.zip › Supplementary_materials.zip!/selected_miRNAs/NoGe_selected_miRNAs/mirdeep_pdf_files/NMRB01000231.1_Notospermus_geniculatus_strain_Ushimado_scaffold231,_whole_genome_shotgun_sequence_7815.pdf]

Provisional ID	:	NMRB01000231.1_Notospermus_geniculatus_strain_Ushimado_scaffold231,_whole_genome_shotgun_sequence_7815
Score total	:	3428.3
Score for star read(s)	:	3.9
Score for read counts	:	3421.1
Score for mfe	:	0.3
Score for randfold	:	0
Score for cons. seed	:	3
Total read count	:	6722
Mature read count	:	5938
Loop read count	:	0
Star read count	:	784

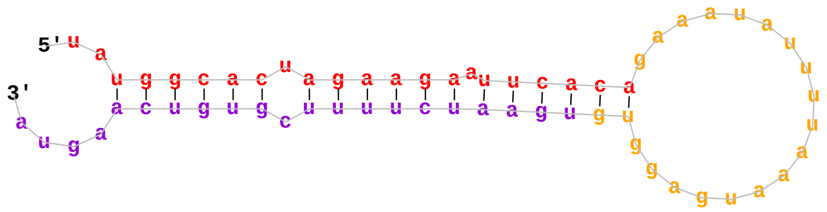

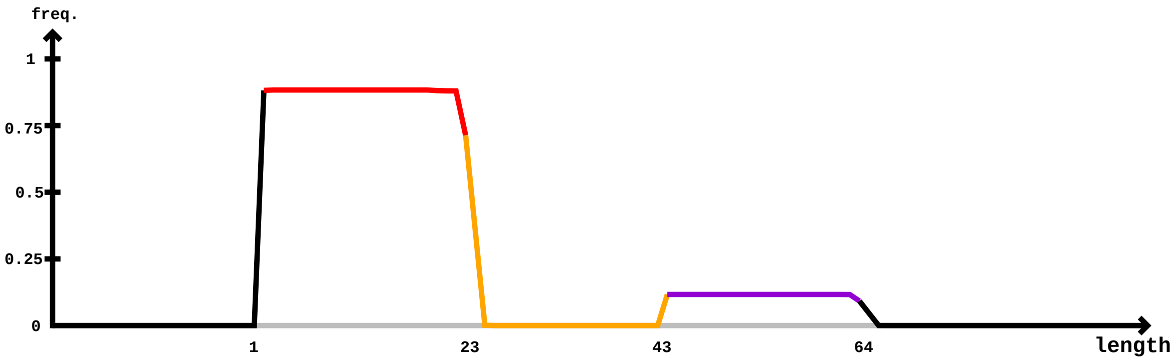


```
                     Mature                                      Star
5'- uuuagaaaguguugucugucuauggcacuagaagaauucacagaaauauuuuaaaugaggugugaaucuuuucgugucaaguacagauugcuccgcuugcuucacgaacuuc  -3'   obs
    uuuagaaaguguugucugucuauggcacuagaagaauucacagaaauauuuuaaaugaggugugaaucuuuucgugucaaguacagauugcuccgcuugcuucacgaacuuc        exp
    ...((.(((((..((((((((.((((((.((((((.((((((...................))))))))))))).)))))))).))))))......))))).))..........   reads    mm    sample
    ....................uauggcacuagaagaauucac.......................................................................   127      0     Fm2
    ....................uauggcacuagaagaauCcac.......................................................................   1        1     Fm2
    ....................uauggcacuagaagaauAcac.......................................................................   4        1     Fm2
    ....................uauggcacuagaagaaAucac.......................................................................   1        1     Fm2
    ....................uauggcacuagaagaauGcaca......................................................................   2        1     Fm2
    ....................uaGggcacuagaagaauucaca......................................................................   1        1     Fm2
    ....................uauggcacuagaagaauCcaca......................................................................   5        1     Fm2
    ....................uauggcUcuagaagaauucaca......................................................................   1        1     Fm2
    ....................uaAggcacuagaagaauucaca......................................................................   1        1     Fm2
    ....................uauggcacuagUagaauucaca......................................................................   1        1     Fm2
    ....................Nauggcacuagaagaauucaca......................................................................   1        1     Fm2
    ....................uauggcacuagaaCaauucaca......................................................................   1        1     Fm2
    ....................uauggcacuagaagaauuUaca......................................................................   1        1     Fm2
    ....................uauggcacuaAaagaauucaca......................................................................   1        1     Fm2
    ....................uauggcacuagaagaaGucaca......................................................................   1        1     Fm2
    ....................uauggcacuagaCgaauucaca......................................................................   1        1     Fm2
    ....................uauggcCcuagaagaauucaca......................................................................   1        1     Fm2
    ....................uauggcacuagaGgaauucaca......................................................................   1        1     Fm2
    ....................uauggcacuGgaagaauucaca......................................................................   1        1     Fm2
    ....................uauggcacuagaagaauAcaca......................................................................   2        1     Fm2
    ....................uauggcacuagaagaauucaca......................................................................   821      0     Fm2
    ....................uauggcacuagaagaaCucaca......................................................................   6        1     Fm2
    ....................uauggcacuagaagaauucacaA.....................................................................   56       1     Fm2
    ....................uauggcacuGgaagaauucacag.....................................................................   1        1     Fm2
    ....................uauggcacuagaagaauucacaC.....................................................................   1        1     Fm2
    ....................uauggcacuagaagaauucacaAa....................................................................   5        1     Fm2
    ..............................................................ugaaucuuCucgugucaagua.............................   1        1     Fm2
    ..............................................................ugaaucuuuucgugucaagua.............................   32       0     Fm2
    ..............................................................ugaaucuuuucgugucaaguaU............................   34       1     Fm2

    ....................uauggcacuagaagaauucac.......................................................................   78       0     Fm1
    ....................uauggcacuagaagaauGcac.......................................................................   1        1     Fm1
    ....................uauggcacuagaagaaGucac.......................................................................   2        1     Fm1
```

Mature **Star**

```
uuuagaaaguguugucugucuauggcacuagaagaauucacagaaauauuuuaaaugaggugugaaucuuuucgugucaaguacagauugcuccgcuugcuucacgaacuuc
....................uauggcacuagaagaauGcaca......................................................................   2     1   Fm1
....................uauggcacuagaagaauucacG......................................................................   1     1   Fm1
....................uauggcacuaAaagaauucaca......................................................................   2     1   Fm1
....................Nauggcacuagaagaauucaca......................................................................   1     1   Fm1
....................uaugAcacuagaagaauucaca......................................................................   1     1   Fm1
....................uauggcacuagaagaauCcaca......................................................................   6     1   Fm1
....................uauggcacuaUaagaauucaca......................................................................   1     1   Fm1
....................uauggcacuagaagaauAcaca......................................................................   3     1   Fm1
....................uauggcacuagaagaaAucaca......................................................................   1     1   Fm1
....................uauggcacuagaagaaGucaca......................................................................   2     1   Fm1
....................uauggcacuGgaagaauucaca......................................................................   1     1   Fm1
....................uauggcacuagaagaauucaca......................................................................   469   0   Fm1
....................uauggcacuagaagaauucacaU.....................................................................   2     1   Fm1
....................uauggcacuagaagaauucacaA.....................................................................   15    1   Fm1
....................uauggcacuagaagaauucacaAa....................................................................   1     1   Fm1
.....................auggcacuagaagaauucac.......................................................................   1     0   Fm1
..............................................................ugaaucuuuucgugucCagu..............................   1     1   Fm1
..............................................................ugaaucuuuuGgugucaagu..............................   1     1   Fm1
..............................................................ugaaucuuuucgugucaagu..............................   8     0   Fm1
..............................................................ugaaCcuuuucgugucaagua.............................   1     1   Fm1
..............................................................ugaaucuuuucgugucaagua.............................   49    0   Fm1
..............................................................Agaaucuuuucgugucaagua.............................   1     1   Fm1
..............................................................ugaaucuuuucgugucGagua.............................   1     1   Fm1
..............................................................ugaaucuuuucgugucaaguaU............................   23    1   Fm1

....................uauggcacuagaagaauuc.........................................................................   3     0   Mm2
....................uauggcacuagaaAaauucac.......................................................................   1     1   Mm2
....................uauggcacuaCaagaauucac.......................................................................   2     1   Mm2
....................uauggcacuagaagaauucUc.......................................................................   1     1   Mm2
....................uauggcUcuagaagaauucac.......................................................................   1     1   Mm2
....................uauggcacuagaagaauGcac.......................................................................   3     1   Mm2
....................uauggcacuagaagaauAcac.......................................................................   1     1   Mm2
....................uauggcacuagaagaaCucac.......................................................................   1     1   Mm2
....................uauggcacuagaagaauucac.......................................................................   468   0   Mm2
....................uauggcacuagaagaauCcac.......................................................................   8     1   Mm2
....................uauggcacuaUaagaauucac.......................................................................   1     1   Mm2
....................uauggcacuagaagaauAcaca......................................................................   11    1   Mm2
....................uauggcacuGgaagaauucaca......................................................................   2     1   Mm2
....................uauggcacuagaagaaCucaca......................................................................   7     1   Mm2
....................uauggcacuaUaagaauucaca......................................................................   3     1   Mm2
....................uauggcacuagaagaauucUca......................................................................   1     1   Mm2
....................uauggcacuagaagaauuUaca......................................................................   2     1   Mm2
....................uaugAcacuagaagaauucaca......................................................................   1     1   Mm2
....................uauggcacuagaagaauCcaca......................................................................   14    1   Mm2
....................uauggNacuagaagaauucaca......................................................................   1     1   Mm2
....................uauggcacuagaagaaAucaca......................................................................   3     1   Mm2
....................uauggcacuagaagaaGucaca......................................................................   4     1   Mm2
....................uauggcacuagaagaauucaca......................................................................   1719  0   Mm2
....................Cauggcacuagaagaauucaca......................................................................   1     1   Mm2
....................uauggcacuagaagaauucacU......................................................................   3     1   Mm2
....................uauggcUcuagaagaauucaca......................................................................   1     1   Mm2
....................uauggcacuagaagaauGcaca......................................................................   10    1   Mm2
....................uauggcacuagaagaUuucaca......................................................................   1     1   Mm2
....................uauggcacuaAaagaauucaca......................................................................   5     1   Mm2
....................uauggcacuagaagaauucacG......................................................................   2     1   Mm2
....................uauggcacuagaagaauucacaU.....................................................................   10    1   Mm2
....................uauggcacuagaagaauucacaA.....................................................................   106   1   Mm2
....................uauggcacuagaagaauucacag.....................................................................   2     0   Mm2
....................uauggcacuagaagaauucacaAa....................................................................   1     1   Mm2
.....................Uuggcacuagaagaauucac.......................................................................   1     1   Mm2
.....................auggcacuagaagaauucaca......................................................................   3     0   Mm2
..............................................................ugaaucuuuucgugucaag...............................   2     0   Mm2
..............................................................ugaaucuuuucgugucaagG..............................   2     1   Mm2
..............................................................ugaaCcuuuucgugucaagu..............................   1     1   Mm2
..............................................................ugaaucuuuucgugucaagu..............................   46    0   Mm2
..............................................................ugaaucuuuucgugucCagu..............................   1     1   Mm2
..............................................................Agaaucuuuucgugucaagua.............................   1     1   Mm2
..............................................................ugaaucuuuucgugucaagua.............................   215   0   Mm2
..............................................................ugaaucuuuucgugucUagua.............................   1     1   Mm2
..............................................................ugaaucuuuucgugucaaguU.............................   1     1   Mm2
```

Mature (uauggcacuagaagaauucaca) — **Star** (ugaaucuuuucgugucaagua)

uuuagaaaguguugucugucuauggcacuagaagaauucacagaaauauuuuaaaugaggugugaaucuuuucgugucaaguacagauugcuccgcuugcuucacgaacuuc			
....ugaaAcuuuucgugucaagua.............................	1	1	Mm2
....ugaaucuuuucgugucaUgua.............................	1	1	Mm2
....ugaaucuuuucgugucaaguaA............................	12	1	Mm2
....ugaaucuuuucgugucaaguaU............................	108	1	Mm2
....ugaaucuuuucgugucaaguaAa...........................	1	1	Mm2
....................uauggcacuagaagaauA..	1	1	Mm1
....................uauggcacuagaagaauu..	12	0	Mm1
....................uauggcacuagaagaauucac...	51	0	Mm1
....................uauggcacuagaagaauAcac...	1	1	Mm1
....................uauggUacuagaagaauucaca..	5	1	Mm1
....................uauggcacuagaagaaCucaca..	1	1	Mm1
....................uauggcacuagaagaauucacU..	5	1	Mm1
....................uauggcacuagaagGauucaca..	1	1	Mm1
....................uauggcacuagaagaauGcaca..	2	1	Mm1
....................uauggcacuagaGgaauucaca..	1	1	Mm1
....................uauggcacuagaagaaGucaca..	2	1	Mm1
....................uauggcacuaUaagaauucaca..	1	1	Mm1
....................uauggcacuagaagaauAcaca..	1	1	Mm1
....................uauggcCcuagaagaauucaca..	1	1	Mm1
....................uauggcacuaAaagaauucaca..	2	1	Mm1
....................uauggcacuagaagaaAucaca..	1	1	Mm1
....................uauggcacuagaagaauucaca..	397	0	Mm1
....................uauggcacuagaagaauuAaca..	1	1	Mm1
....................uauggcacuagaagaauucacaA...	42	1	Mm1
.....................auggcacuagaagaauucaca..	1	0	Mm1
....ugaaucuuuucgugucaagu..............................	39	0	Mm1
....ugaaucuuuucgugucaagua.............................	44	0	Mm1
....ugaaucuuuucgugucaaguaU............................	10	1	Mm1
....................uauggcacuagaagaauu..	2	0	Fm3
....................uauggcacuagaagaauuc...	1	0	Fm3
....................uauggcacuagaagaauuca..	1	0	Fm3
....................uauggcacuagaUgaauucac...	1	1	Fm3
....................uauggcacuagaagaauCcac...	1	1	Fm3
....................uauggcacuaUaagaauucac...	1	1	Fm3
....................uauggcacuagaagaaAucac...	2	1	Fm3
....................uCuggcacuagaagaauucac...	1	1	Fm3
....................uauggcacuagaagaauAcac...	1	1	Fm3
....................uauggcacuagaagaauGcac...	3	1	Fm3
....................uauggcacuagaagaauucac...	252	0	Fm3
....................uauggcacuagCagaauucac...	1	1	Fm3
....................uauggcacuaAaagaauucac...	2	1	Fm3
....................uauggcacuaCaagaauucac...	1	1	Fm3
....................uauggcacuagaagaaGucac...	1	1	Fm3
....................uauggcacuagaagaauucaca..	640	0	Fm3
....................Nauggcacuagaagaauucaca..	1	1	Fm3
....................uauggcacuagaagaauAcaca..	6	1	Fm3
....................uauggcacuagaagaaAucaca..	3	1	Fm3
....................uauggcacuagUagaauucaca..	1	1	Fm3
....................uauggcacuagaagaauucacU..	4	1	Fm3
....................Aauggcacuagaagaauucaca..	1	1	Fm3
....................uauggcacuagaagaauGcaca..	5	1	Fm3
....................uauggcacuaUaagaauucaca..	1	1	Fm3
....................Cauggcacuagaagaauucaca..	5	1	Fm3
....................uauAgcacuagaagaauucaca..	1	1	Fm3
....................uauggNacuagaagaauucaca..	1	1	Fm3
....................uauggcacuagaagaauCcaca..	5	1	Fm3
....................uauggcacGagaagaauucaca..	1	1	Fm3
....................uauggcacuagaagaaGucaca..	3	1	Fm3
....................uauggcacuagaagaauuUaca..	1	1	Fm3
....................uauggcacuagaagaaCucaca..	3	1	Fm3
....................uauggcacuagaagaauucacaA...	10	1	Fm3
....................uauggcacuagaagaauucacaU...	3	1	Fm3
.....................auggcacGagaagaauucac...	1	1	Fm3
.....................auggcacuagaagaauucaca..	2	0	Fm3
.....................auggcacuagaagaauucacaga..	1	0	Fm3
....ugaaucuuuucgugucaagu..............................	50	0	Fm3
....ugaaucuuuucgugucUagu..............................	1	1	Fm3
....ugaaucuuuucgugucaagua.............................	47	0	Fm3

```
                    Mature                                      Star
uuuagaaaguguugucugucuauggcacuagaagaauucacagaaauauuuuaaaugaggugugaaucuuuucgugucaaguacagauugcuccgcuugcuucacgaacuuc
..............................................................ugaaucuuuucguCucaagua.............................  1    1  Fm3
..............................................................ugaaucuuuucgugucaaguaU............................  12   1  Fm3

....................uauggcacuagaagaauu..........................................................................  1    0  Mm3
....................uauggcacuagaagaauuc.........................................................................  1    0  Mm3
....................uauggcacuagaagaauuca........................................................................  1    0  Mm3
....................uauggcacuagaagaauucac.......................................................................  94   0  Mm3
....................uauggcacuagaagaaGucac.......................................................................  1    1  Mm3
....................uauggcacuagaagaauCcac.......................................................................  1    1  Mm3
....................uauggcacuagaagaauuUaca......................................................................  1    1  Mm3
....................uauggcacuagaagaaAucaca......................................................................  2    1  Mm3
....................uauggcacuagaagaauucGca......................................................................  1    1  Mm3
....................uauggcacuagaagaauucaca......................................................................  283  0  Mm3
....................uauggcacuagaagaauucacU......................................................................  1    1  Mm3
....................uauggcacuaCaagaauucaca......................................................................  1    1  Mm3
....................uauggcacuagaagaauucacaU.....................................................................  1    1  Mm3
....................uauggcacuagaagaauucacaA.....................................................................  10   1  Mm3
....................uauggcacuagaagaauucacaAa....................................................................  1    1  Mm3
..............................................................ugaaucuuuucgugucaagu..............................  12   0  Mm3
..............................................................ugaaucuuuucgugucUagu..............................  1    1  Mm3
..............................................................ugaaucuuuucgugucCagu..............................  1    1  Mm3
..............................................................ugaaucuuuucgugucaagua.............................  12   0  Mm3
..............................................................ugaaucuuuucgugucaaguaA............................  4    1  Mm3
..............................................................ugaaucuuuucgugucaaguaU............................  5    1  Mm3
```
